# Micro-/Nano-Structures Fabricated by Laser Technologies for Optoelectronic Devices

**DOI:** 10.3389/fchem.2021.823715

**Published:** 2021-12-16

**Authors:** Jian Yi, Hao Zhou, Wei-Hua Wei, Xing-Chen Han, Dong-Dong Han, Bing-Rong Gao

**Affiliations:** State Key Laboratory of Integrated Optoelectronics, College of Electronic Science and Engineering, Jilin University, Changchun, China

**Keywords:** micro-/nano-structures, laser technologies, photodetector, photovoltaic cell, light-emitting diode

## Abstract

Due to unique optical and electrical properties, micro-/nano-structures have become an essential part of optoelectronic devices. Here, we summarize the recent developments in micro-/nano-structures fabricated by laser technologies for optoelectronic devices. The fabrication of micro-/nano-structures by various laser technologies is reviewed. Micro-/nano-structures in optoelectronic devices for performance improvement are reviewed. In addition, typical optoelectronic devices with micro-nano structures are also summarized. Finally, the challenges and prospects are discussed.

## Introduction

There are many animals and plants using unique micro-/nano-structures to improve their environmental adaptability (Han et al., 2016; Han et al., 2020a; Cao et al., 2020). For example, micro-/nano-structures on a lotus leaf and taro surface exhibit superhydrophobic properties (Zhang et al., 2012b; Wang et al., 2021b; Lv et al., 2021). Grating-like structures on butterfly wings trap light, leading to the colorful butterfly wing (Wang et al., 2012; Jiang et al., 2016; Zou et al., 2020). Learning from nature, micro-/nano-structures have been adopted in the various functional devices for broad applications (Han et al., 2019; Zhang et al., 2019; Zhang et al., 2021). Therefore, many researchers have focused on the fabrication and application of micro-nano structures (Zhang et al., 2012a; Han et al., 2015; Liu et al., 2021). Mainly, due to unique optical and electrical properties, micro-/nano-structures have become an essential part of optoelectronic devices.

Laser fabrication technologies show high efficiency, high precision, and low thermal effect (Li et al., 2020a; Fu et al., 2020; Ma et al., 2020; You et al., 2020; Fu et al., 2021). Laser technologies can be used to fabricate micro-/nano-structures by the interaction between laser and materials (Liu et al., 2019; Han et al., 2020b; Liu et al., 2020; Wang et al., 2021a). Especially, ultrafast lasers can fabricate broadband, transparent anti-reflection surfaces, which promote the performance of optoelectronic devices by enhancing the light absorption or introducing surface plasmon-polariton (Zhang et al., 2010; Liapis et al., 2017; Jia et al., 2020).

In this review, we summarize recent progress on micro-/nano-structures fabricated by laser technologies. Typical light trapping mechanism and surface plasmon-polariton of the micro-nano structure are discussed. Then, we outlined the typical applications, including photodetectors, photovoltaic cells, organic light-emitting devices, etc. Finally, the challenges and prospects are discussed.

## Mechanism

Introducing micro-/nano-structures inside or outside the devices can improve optoelectronic devices’ performance (Ma and Cui, 2020; Na and Chew, 2020; Chen et al., 2021). Inspired by the moth-eye structure, the reflectivity is reduced by introducing micro-/nano-structures. Mainly, the light will be internally reflected many times inside the structure to form a “light trap” (Zhang et al., 2020a; Otte and Denz, 2020; Yang et al., 2021). As a result, the existence of micro-/nano-structures can improve the light absorption capacity of the optoelectronic device. Moreover, the efficiency of optoelectronic devices can be enhanced by surface plasmon-polariton (Eaton et al., 2016; Li et al., 2020b; Zhang et al., 2020b).

## Optoelectronic Devices

### Photodetector

**FIGURE 1:**
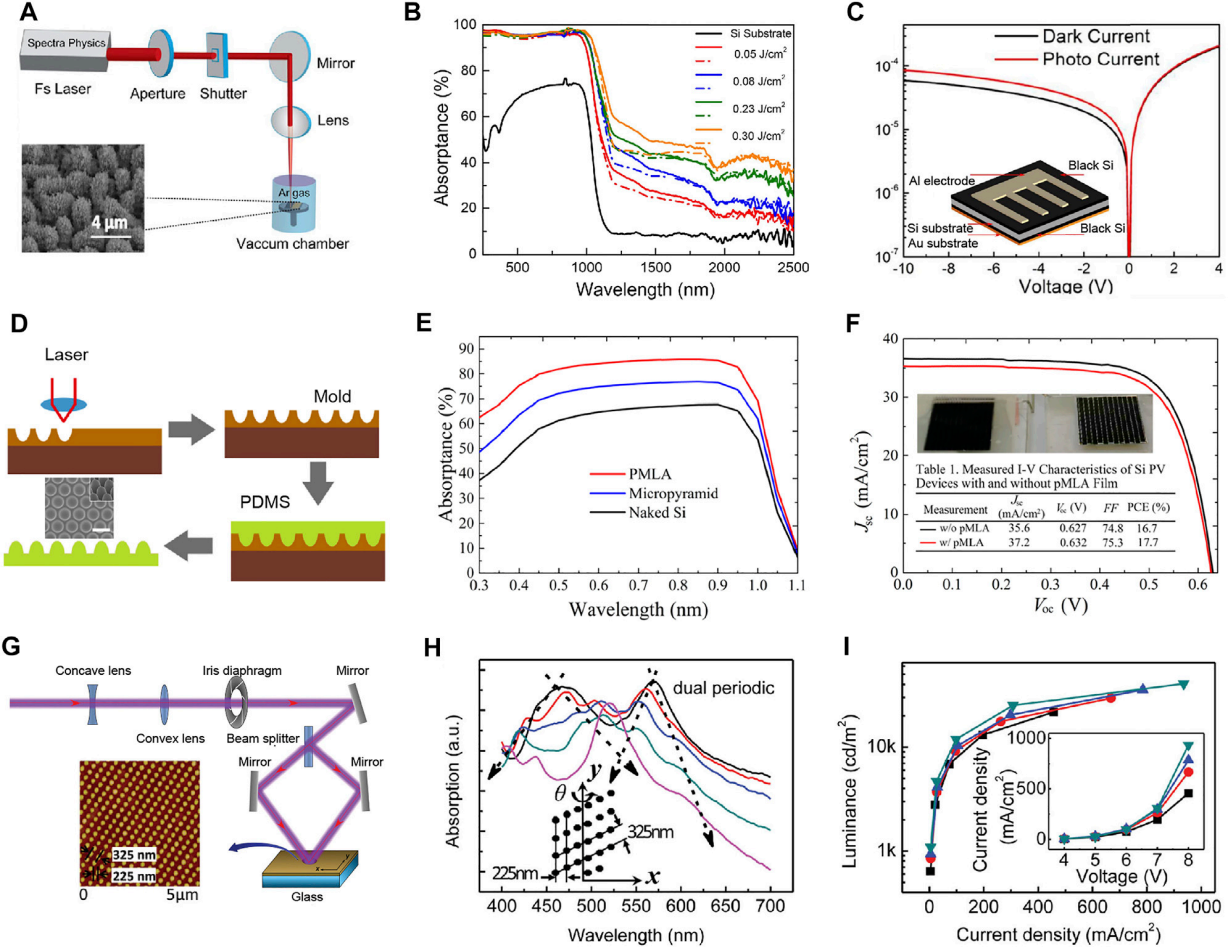
Micro-/nano-structures fabricated by laser technologies for optoelectronic devices. **(A)** Femtosecond laser manufacturing system. Reproduced from (Yu et al., 2017) with permission of IEEE. **(B)** The corresponding absorptivity of nitrogen-doped silicon substrate. **(C)** Infrared photodetection performance. Reproduced from (Li et al., 2018) with permission of IEEE. **(D)** The preparation process of PDMS convex PMLA film. **(E)** Simulated absorption spectra of films. **(F)** Solar cell performance. Reproduced from (Fang et al., 2018) with permission of American Chemical Society. **(G)** Schematic diagram of laser two-beam interferes processing. Reproduced from (Bi et al., 2013; Jiang et al., 2014) with permission of WILEY-VCH. **(H)** Absorption spectra for WOLEDs. **(I)** Performance of WOLED. Reproduced from (Bi et al., 2013) with permission of WILEY-VCH.

Silicon material plays an important role in silicon-based optoelectronic integrated devices preparation. Take photodetectors as an example, the bandgap of silicon material is around 1.1–1.3 eV, limiting silicon material for infrared radiation (IR) photodetection. Therefore, many efforts, such as ion implantation or structural defects, have been developed to extend the absorption band of silicon. As a pioneer, Zhao’s group (Li et al., 2017; Yu et al., 2017; Li et al., 2018) fabricated supersaturated silicon material with nitrogen, sulfur, and Au by femtosecond laser ablation (Figure 1A). After the femtosecond laser ablation in nitrogen (N_2_) atmosphere, the surface silicon material evolved into a bead-like micro-/nano-structures with a height of 3∼4 μm and a distance of 3∼4 μm (Li et al., 2018). Micro-/nano-structures are beneficial for a stronger light trapping effect. Compared with the initial silicon material, laser-treated N-doped silicon material has a broader absorption (0.25–2.5 μm) and higher absorptivity (Figure 1B). The inset of Figure 1C is the device structure of the laser-treated silicon-based IR photodetector. The photo responsivity is 5.3 mA/W (V = 10 V).

### Photovoltaic Cell

Photovoltaic cells convert sunlight to electric energy. Usually, light utilization efficiency is very low due to the reflection loss. To solve this problem, various anti-reflection structures have been designed. For example, Fang et al. proposed a 100% relative packing density film for enhancing photovoltaic cells performance (Fang et al., 2018). As shown in Figure 1D, direct-write ultraviolet (UV) laser photolithography system was employed to fabricate a paraboloidal concave photoresist pattern (master mold). Then polydimethylsiloxane (PDMS) was spin-coated onto the master mold. After thermally cured, structured PDMS was separated from the master mold for further use. Figure 1E is the simulated absorption spectra of films. Si substrate with paraboloidal microlens array (PMLA) film shows the highest absorption due to the suppressing reflection. It is worth noting that PMLA antireflective (AR) film indicates superhydrophobicity and self-cleaning ability. Finally, the short-circuit current density increases from 35.6 to 37.2 mA/cm^2^ after integrating the PMLA AR film (Figure 1F). Instead of integrating the AR film on the photovoltaic cells surface, structured photoelectrodes or active layers have been fabricated by femtosecond laser ablation, interference, or laser-induced periodic surface structures for photocurrent enhancement (Zhang et al., 2015; Cui et al., 2016; Soldera et al., 2016).

### Light-Emitting Diode

Bi et al. (2013) demonstrated white organic light-emitting diodes (WOLEDs) with broadband excitation by introducing two-dimensional gratings. As shown in Figure 1G, the grating structures were prepared by two-beam laser interference (Guo et al., 2012; Jiang et al., 2014; Yan et al., 2015). Introducing dual-period corrugations into the WOLED metal electrodes achieves broadband absorption (Figure 1H). In addition, broadband SPP modes lead to broadband light extraction. Significantly, broadband light extraction deeply affects the WOLEDs performance (Figure 1I). Compared with traditional planar devices, the current efficiency is increased by 37%, and the external quantum efficiency is increased by 48%. Recently, combining laser interference lithography and reactive ion etching, Ju et al. proposed flexible OLEDs with light extraction structure for optical efficiency improvement (Lee et al., 2019; Kim et al., 2020).

## Conclusion and Outlook

This minireview summarizes recent progress on micro-/nano-structures fabricated by laser technologies for optoelectronic devices. The existence of micro-/nano-structures can improve the light absorption capacity and the efficiency of optoelectronic devices. Typical optoelectronic devices have been successfully designed and demonstrated the critical role of micro-/nano-structures. Significantly, new photoelectric applications, such as photoelectric dichroism, have been proposed and fabricated by laser technology based on various materials (Drevinskas et al., 2015; Jiang et al., 2020; Kuroiwa and Tatsuma, 2020; Zou et al., 2021a; Zou et al., 2021b; Xuan et al., 2021). Although successful works have demonstrated the distinguish characters, the efficiency of laser processing materials needs to improve, which benefits device preparation efficiency. With the rapid development of nanofabrication technology, advanced fundamental theories, new structural design, micro-/nano-structures will improve devices performances.
